# Torticollis in incomplete Kawasaki disease: a case of atlantoaxial rotatory fixation

**DOI:** 10.1093/bjrcr/uaae044

**Published:** 2024-11-22

**Authors:** Keisho Ryu

**Affiliations:** Department of Orthopaedic Surgery, Tokyo Metropolitan Toshima Hospital, Tokyo 173-0015, Japan

**Keywords:** atlantoaxial subluxation, Grisel’s syndrome, Kawasaki disease, cervical lymphadenopathy, torticollis

## Abstract

Various respiratory, musculoskeletal, gastrointestinal, neurological, and urinary complications have been reported in Kawasaki disease. Here, we describe a rare case of atlantoaxial rotatory fixation (AARF) associated with incomplete Kawasaki disease. The case is of a healthy 4-year-old Japanese boy who had a high-grade fever, lymphadenopathy, and torticollis diagnosed with incomplete Kawasaki disease. Intravenous high-dose immunoglobulin and oral aspirin quickly resolved his fever and improved his lymphadenopathy, but torticollis remained. On orthopaedic examination, torticollis was observed with a marked restriction of rotation, and an open-mouth anteroposterior cervical radiograph and a CT scan confirmed rotational dislocation at the dens axis (AARF). Cervical collar fixation was immediately started, and the torticollis gradually normalized within a week. AARF is defined as torticollis due to dislocation or subluxation of the atlantoaxial joint. The diagnosis of AARF is difficult with routine plain cervical radiographs in 2 directions alone, and an additional cervical open-mouth anteroposterior radiograph and a CT scan aid the diagnosis. AARF associated with Kawasaki disease is uncommon, and only 24 cases have been reported in the literature. AARF may occur in Kawasaki disease patients with cervical lymphadenopathy. Still, torticollis is often transient and may not be recognized or ignored by family doctors and paediatricians. Reduction of the atlantoaxial joint can often be achieved spontaneously or with conservative treatment such as a collar or neck traction, but treatment is difficult if the diagnosis is delayed. Therefore, family doctors and paediatricians need to suspect the onset of AARF if torticollis is observed during treatment for Kawasaki disease, perform plain cervical radiographs including open-mouth anteroposterior view and a CT scan of the cervical spine, and have orthopaedists immediately intervene to avoid invasive surgery.

## Introduction

Kawasaki disease is a frequent acute systemic vasculitis of children aged 6 months to 5 years old, accompanied by fever, conjunctival injection, erythema of lips and oral mucosa, exanthema, swellings in extremities, and cervical lymphadenopathy.^1^^,^^2^ Various respiratory, musculoskeletal, gastrointestinal, neurological, and urinary complications have been reported, and careful initial management of evolving cardiovascular abnormalities leading to coronary artery aneurysms is essential.^1^^,^^3^ However, there are few reports of torticollis and atlantoaxial rotatory fixation (AARF) as complications.^1^^,^^2^ Here, we describe a rare case of AARF associated with incomplete Kawasaki disease.

## Clinical presentation

A healthy 4-year-old Japanese boy without any past family history had a high-grade fever. He was prescribed an oral cephalosporin, cefditoren-pivoxil (9 mg/kg/day), with a diagnosis of group A streptococcal infection (day 1 of illness). On day 2 of illness, a swelling of his left neck appeared, and laboratory findings showed a high white blood cell count (WBC) (26.3 × 10^9^/L) and C-reactive protein level (88.4 mg/L). He was hospitalized in the paediatric ward with the diagnosis of acute purulent lymphadenitis, and intravenous ampicillin/sulbactam (10 mg/kg/day) was administered immediately. Despite systemic administration of antibiotics, his fever and cervical lymphadenopathy did not improve, and torticollis appeared on day 4. Echocardiography showed tricuspid regurgitation but no apparent coronary artery lesions. On day 7, he was diagnosed with incomplete Kawasaki disease according to the clinical statements and guidelines of the American Heart Association,^3^ which met 2 of the 6 Kawasaki disease criteria (erythema and oedema of the hands and feet in acute phase, cervical lymphadenopathy) and 4 laboratory findings (anaemia for age, platelet count of ≥450 × 10^9^/L after the seventh day of fever, elevated ALT level and WBC count of ≥15.0 × 10^9^/L). Intravenous high-dose immunoglobulin (2 g/kg) and oral aspirin (30 mg/kg/day) were commenced, quickly resolving his fever and improving his lymphadenopathy, but torticollis remained.

## Investigations/imaging findings

**Figure 1.**
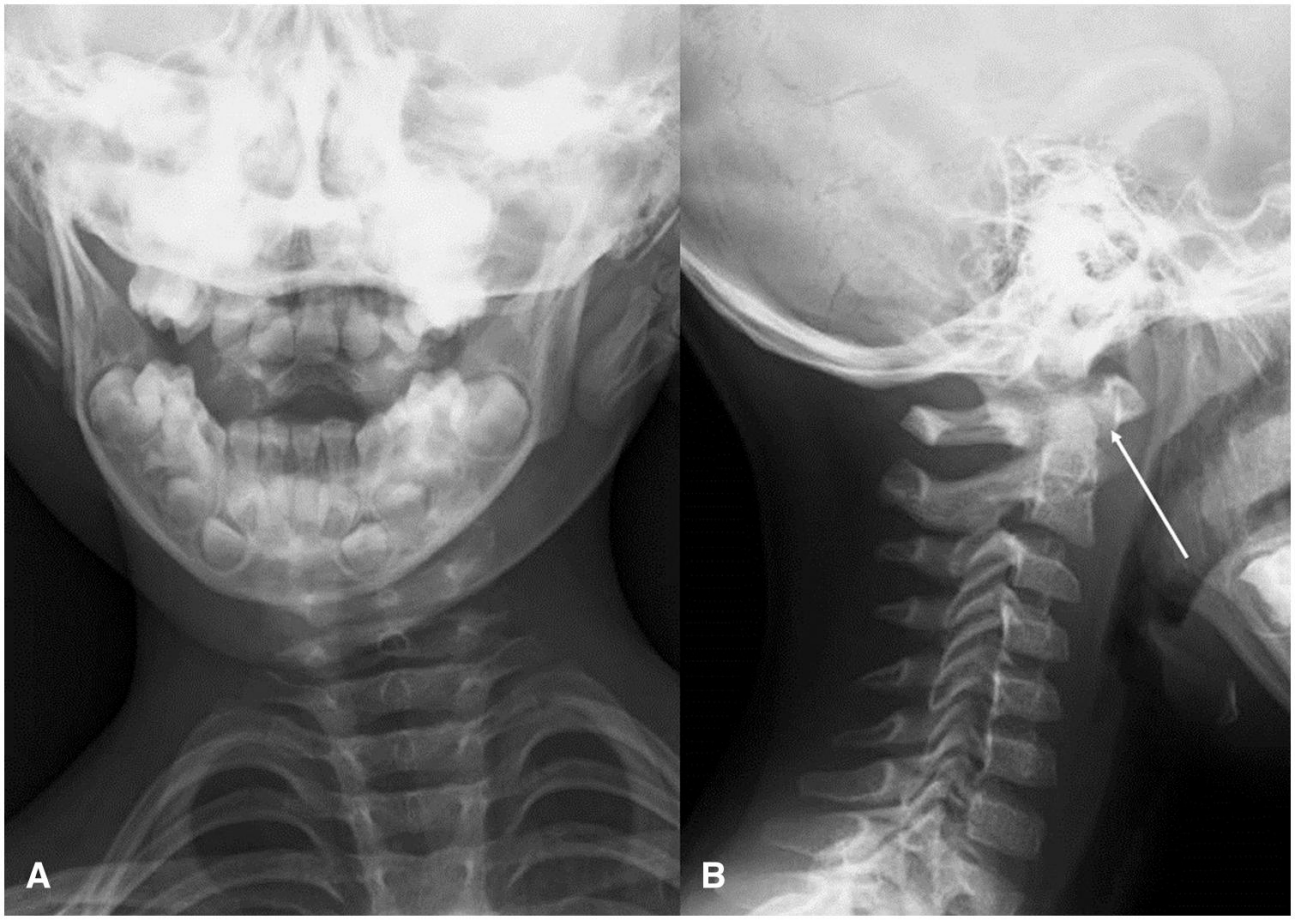
Routine plain cervical radiographs in 2 directions. (A) Anteroposterior view of the cervical spine showing head tilting. The atlantoaxial joint is difficult to evaluate in this image. (B) The lateral view. Atlanta-dental interval (arrow) was within the normal range at 4.4 mm.

**Figure 2.**
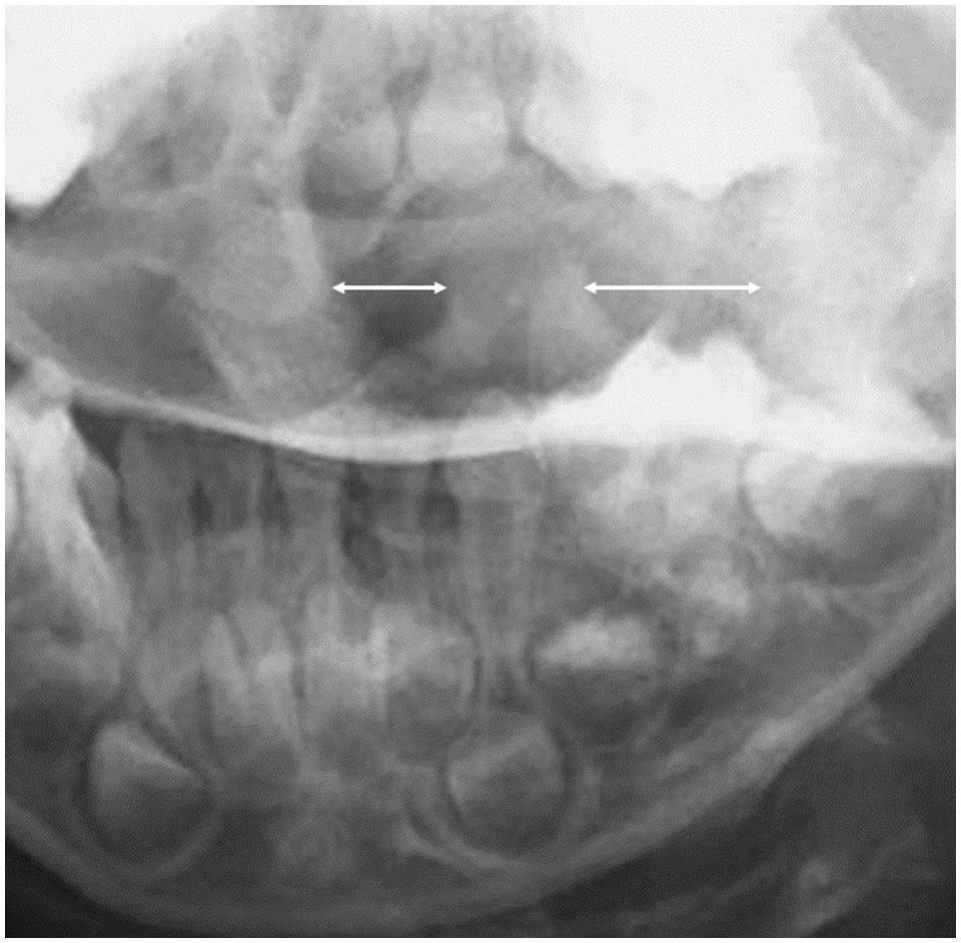
Open-mouth anteroposterior view of the cervical spine showing asymmetry of the axis in the atlantoaxial joint.

**Figure 3.**
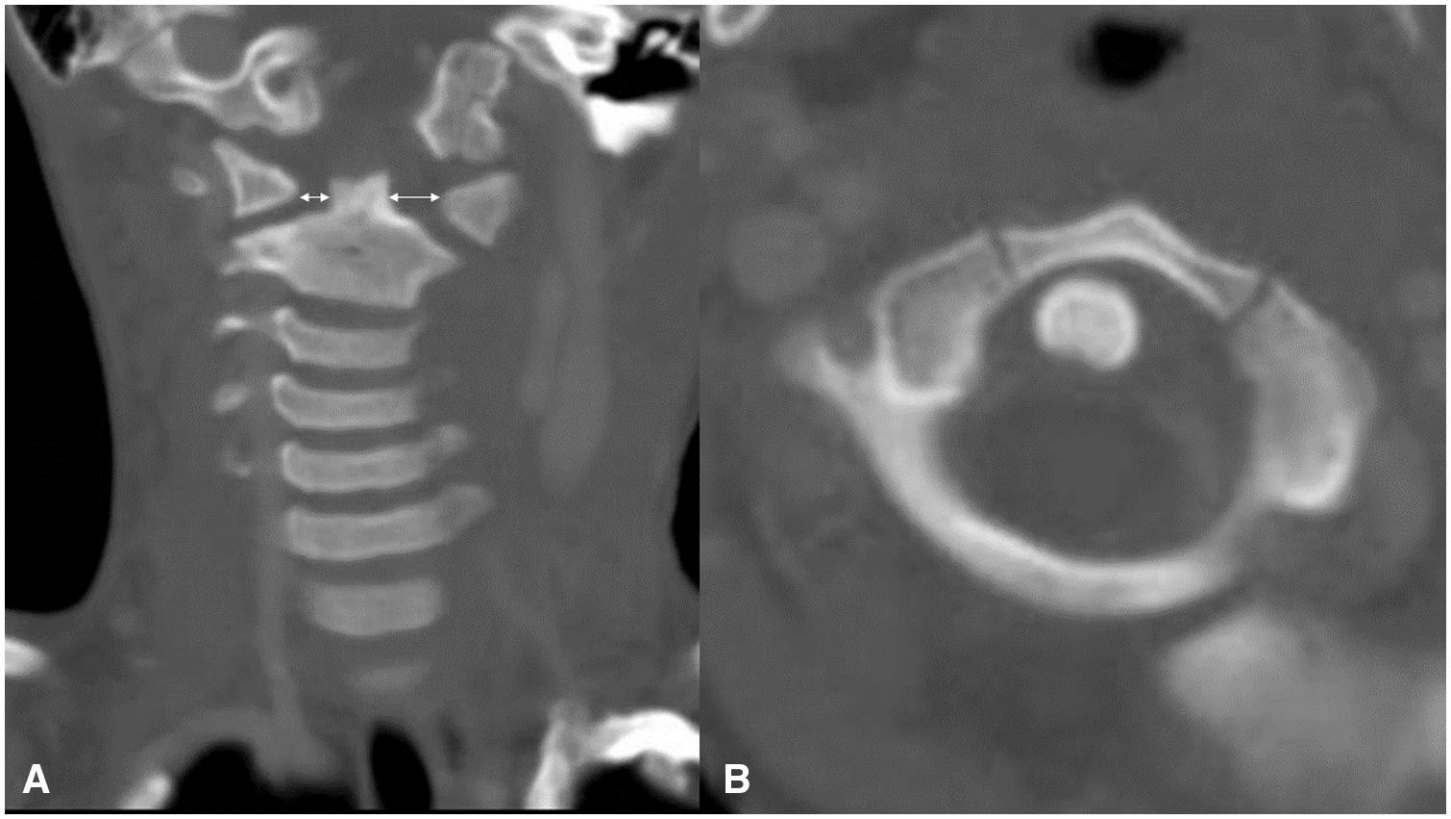
CT scan of the cervical spine. (A) Coronal CT image showing a gap on the left C1-C2 joint. (B) Axial CT image showing atlantoaxial subluxation of Fielding classification type I.

Plain cervical radiographs in 2 directions, anteroposterior and lateral views, and an orthopaedic referral were made. An anteroposterior view of the cervical spine (Figure 1A) showed tilting of the head, and a lateral view of the cervical spine (Figure 1B) showed a normal Atlanta-dental interval (arrow) at 4.4 mm. On orthopaedic examination, torticollis was observed with a marked restriction of rotation, and an extra cervical radiograph (open-mouth anteroposterior view) and a CT scan were performed to investigate atlantoaxial abnormalities. An open-mouth anteroposterior view of the cervical spine (Figure 2) showed asymmetry of the axis in the atlantoaxial joint. CT scan of the cervical spine using bone windowing technique revealed that the odontoid peg was no longer lying centrally and showed gapping on the left C1-C2 articulation on coronal view (Figure 3A). Axial view (Figure 3B) confirmed rotational dislocation at the dens axis (AARF, Fielding classification type I: simple rotation displacement).

## Differential diagnosis

Traumatic AARF.

Spasmodic torticollis.

## Treatment, outcome, follow-up

Once cervical radiographs and CT images diagnosed AARF on day 9 of illness, conservative therapy with cervical collar fixation was immediately started. The torticollis position gradually improved from day 10 and normalized on day 16. The patient had complete symptomatic relief with no neurological sequelae or recurrence in his 2-year follow-up.

## Discussion

AARF is defined as torticollis due to dislocation or subluxation of the atlantoaxial joint by Fielding and Hawkins in 1977.^5^ Most cases occur in childhood and show symptoms of cervical pain, stiffness, and a typical cock robin position. It often develops after a minor trauma, an upper respiratory tract infection (known as Grisel’s syndrome), or oral/pharyngeal surgery.^4^^,^^5^ The pathogenesis of AARF is unclear, but 2 hypotheses have been proposed. The first is increased ligament laxity between C1-C2 and the transverse ligament in children, and the second is spasm caused by an inflammatory process.^2^ The diagnosis of AARF is difficult with routine plain cervical radiographs in 2 directions alone, and an additional cervical open-mouth anteroposterior radiograph may aid in the diagnosis. Furthermore, CT scans are useful for diagnosing and classifying AARF.^5^ Fielding classification assessing the direction and extent of atlas rotation is the most widely accepted and used method in describing AARF. Details of the Fielding classification are as below. Type I: simple rotational displacement, type II: anteroposterior displacement of 3-5 mm, type III: anteroposterior displacement of more than 5 mm, and type IV: posterior displacement. Reduction of the atlantoaxial joint can often be achieved spontaneously or with conservative treatment such as a collar or neck traction, but treatment is difficult if the diagnosis is delayed.^5^ In those cases, rigid immobilization with a halo brace or surgical procedures such as C1-C2 fusion may be required.^4^ Mahr et al^4^ recommended a comprehensive step-by-step treatment algorithm to achieve and retain anatomic reduction of the atlantoaxial joint. They recommend “wait and see” for 3 days, and analgesia immobilization would continue until symptom relief (step 1). If the AARF persists for more than 3 days, they aim for a closed reduction of the atlantoaxial joint under anaesthesia. After a closed reduction of the atlantoaxial joint, they recommended immobilization of the cervical spine by a rigid cervical collar for 4 weeks (step 2). In cases of recurrent dislocation, they performed a second attempt of closed reduction and external fixation with a halo brace, providing more stability (step 3). For rare cases of persistent instability and recurrent dislocations despite using a halo-brace, posterior instrumentation or C1-C2 fusion may be performed. These invasive surgeries should be reserved for patients with recurrent dislocations and failure of all other, less invasive therapeutic options (step 4).^4^ In a 3-dimensional CT image, the lateral inclination of the atlas to the axis ≥20° has been reported as a factor inhibiting reduction, and the deformity of the C2 facet is considered to be a factor for recurrence after reduction.^5^ Although the Fielding classification is the most common classification for AARF, there is a limited correlation between the Fielding classification and re-dislocation rate after conservative treatment or long-term outcome.^4^

AARF associated with Kawasaki disease is uncommon despite the presence of inflammatory processes at the neck, which might cause ligament hypermobility, with distension and abnormal laxity of ligaments surrounding the neck articulation and sternomastoid spasm, with only 24 cases have been reported in the literature.^1^^,^^2^ Liu et al^1^ stated that AARF is a rare complication of Kawasaki disease with an incidence of 0.6% (10/1582), predominantly affecting older female children in their population. They described the presence of lymphadenopathy as the initial and primary symptom, and patients improved with conservative therapy for AARF.^1^ Our case was relatively old, at 4 years, and presented with cervical lymphadenopathy, which improved with conservative treatment and was similar to the previous case reports except for gender. AARF may occur more frequently in Kawasaki disease patients with cervical lymphadenopathy, but the phenomenon of torticollis is often transient and may not be recognized or ignored by family doctors and paediatricians.^1^ To elucidate AARF in Kawasaki disease, it is necessary to inform family doctors and paediatricians to accumulate and analyse more cases.

AARF is a rare complication in Kawasaki disease, but treatment is difficult if the diagnosis is delayed. Therefore, family doctors and paediatricians need to suspect the onset of AARF if torticollis is observed during treatment for Kawasaki disease, perform plain cervical radiographs including open-mouth anteroposterior view and a CT scan of the cervical spine, and have orthopaedists immediately intervene to avoid invasive surgery.

## Learning points

- Suspect the onset of atlantoaxial rotatory fixation (AARF) in the case of torticollis during treatment for Kawasaki disease.
- Reduction of the atlantoaxial joint can often be achieved spontaneously or with conservative treatment such as a collar or neck traction, but treatment is difficult if the diagnosis is delayed. In those cases, rigid immobilization with a halo brace or surgical procedures may be required.
- For the diagnosis of AARF, it is recommended to add an open-mouth anteroposterior cervical radiograph and a CT scan of the cervical spine, as plain cervical radiographs in 2 directions alone may be insufficient for accurate diagnosis.
